# The perception of Romanian mental health professionals on electroconvulsive therapy

**DOI:** 10.1192/j.eurpsy.2024.1461

**Published:** 2024-08-27

**Authors:** C. E. Anghel, C. Băcilă

**Affiliations:** ^1^Faculty of Medicine, The “Lucian Blaga” University; ^2^Scientific Research Collective in Neurosciences - The “Dr. Gheorghe Preda” Clinical Hospital of Psychiatry, Sibiu, Romania

## Abstract

**Introduction:**

The journey for the electroconvulsive therapy began in 1938, when convulsive seizures induced by electrical stimulus were used, for the first time, in the therapy of patients diagnosed with Schizophrenia. Over the time, this therapy remains an important one, due to its applicability and necessity in the therapeutic management of patients with psychiatric pathology.

**Objectives:**

Electroconvulsive therapy has evolved as a technique, nowadays being applied under induced intravenous anesthesia with the administration of oxygen on the mask, and from 2001, the sinus electrical stimulus has been replaced by the one in the form of a short pulse, upon the recommendation of professional organizations, in order to increase its therapeutic effectiveness. However, this form of therapy continues to be stigmatized, largely due to the way it is presented in the mass media. The objective of this work was to analyze how mental health professionals perceive electroconvulsive therapy.

**Methods:**

We conducted a study in which we used a questionnaire applied to the Romanian professionals in the field of mental health.

**Results:**

The results were analyzed in accordance with the objective of the study.

**Conclusions:**

Through this analysis we wanted to understand how electroconvulsive therapy is seen through the eyes of mental health professionals and to identify those aspects that can help us in carrying out information programs, with a major impact on mental health, in order to reduce stigma forasmuch the therapeutic benefits of electroconvulsive therapy outweigh the possible risks.

**Disclosure of Interest:**

None Declared

